# Optimizing exercise interventions in prostate cancer care: methodological considerations and future directions

**DOI:** 10.1097/JS9.0000000000003260

**Published:** 2025-08-25

**Authors:** Zhengyan Wang, Hongjin Shi, Haifeng Wang

**Affiliations:** a Department of Urology, Honghe Hospital Affiliated to Kunming Medical University/South Yunnan Central Hospital of Yunnan Province (The First People’s Hospital of Honghe Hani and Yi Autonomous Prefecture), Honghe, China; b Kunming Medical University, Kunming, China; c Department of Urology, the Second Affiliated Hospital of Kunming Medical University, Kunming, China

*Dear Editor,*

We reviewed with great interest the meta-analysis by Nader *et al*^[^1^]^ in the *International Journal of Surgery*, which evaluated the effects of aerobic and resistance training on fatigue, quality of life (QoL), and physical activity in prostate cancer (PCa) patients. The study demonstrated that these exercises significantly improved QoL but did not substantially reduce fatigue or enhance physical activity. These findings underscore the therapeutic role of exercise in PCa management. This letter follows the TITAN Guidelines 2025 (regarding AI use transparency) and confirms zero utilization of AI tools in its preparation^[^2^]^.

Common PCa treatments—surgery, radiotherapy, and endocrine therapy—have improved survival rates^[^3^]^, yet they often cause adverse effects, including fatigue, reduced physical activity, and diminished QoL. Prolonged endocrine therapy may induce cancer-related fatigue (CRF), while prostatectomy can impair pelvic floor function, leading to urinary incontinence. Targeted exercise interventions may mitigate these effects through multiple mechanisms, such as antioxidant effects, metabolic regulation, body composition improvement, anti-inflammatory responses, and immune system enhancement^[^4^]^.

This study exhibits several methodological limitations that warrant further refinement. Primarily, the potential moderating effects of disease stage (localized versus metastatic PCa) and treatment modalities (radiation therapy versus androgen deprivation therapy) on exercise intervention efficacy remain insufficiently evaluated. To address this, stratified regression or subgroup analyses should be employed to elucidate heterogeneity in treatment responses across clinically distinct patient populations, with particular emphasis on stratification by TNM stage, Gleason score, and therapeutic regimen. Furthermore, while the study delineates exercise intervention types, it omits a meta-regression analysis of critical exercise parameters—such as intensity, frequency, and duration. Future investigations should incorporate a dose-response analysis, specifically gexamining the association between total weekly exercise volume and clinical outcomes. Regarding outcome interpretation, the observed discordance between QoL improvements and fatigue symptom alleviation necessitates further scrutiny. Given the multidimensional structure of the EORTC QLQ-C30 scale, independent trend analyses of functional subscales (e.g., emotional and social functioning) and symptom subscales (e.g., fatigue, pain) are recommended to identify the precise determinants of QoL enhancement. The superior performance of the control group in the 400-meter walk test may reflect baseline functional disparities or suboptimal exercise prescription intensity. From a translational perspective, the current findings lack clinically actionable recommendations. Thus, evidence-based, stage-specific exercise prescriptions should be formulated. Future research should prioritize the following: (1) investigating differential exercise effects on fatigue subtypes, (2) assessing the role of digital health technologies (e.g., wearable devices) in enhancing adherence, and (3) identifying exercise-responsive biomarkers. Lastly, publication bias should be quantitatively assessed using the Egger test, with corresponding correction coefficients reported to strengthen methodological rigor.

This study provides a valuable framework for investigating integrated exercise interventions and key challenges in cancer care, particularly fatigue and physical activity limitations. We anticipate future large-scale, multicenter clinical trials to strengthen the evidence base for clinical practice.

## Data Availability

Not applicable.

## References

[1] Nader S Massoud A Al-Obeidat F. Impact of aerobic and resistance training on fatigue, quality of life, and physical activity in prostate cancer patients: a systematic review and meta-analysis. Int J Surg 2024;110:6170–81. 38224407 10.1097/JS9.0000000000000982 PMC11487001

[2] Agha RA Mathew G Rashid R. Transparency in the reporting of Artificial INtelligence – the TITAN guideline. Prem J Sci 2025;10:100082–85.

[3] Monga U Garber SL Thornby J. Exercise prevents fatigue and improves quality of life in prostate cancer patients undergoing radiotherapy. Arch Phys Med Rehabil 2007;88:1416–22. 17964881 10.1016/j.apmr.2007.08.110

[4] Torti DC Matheson GO. Exercise and prostate cancer. Sports Med 2004;34:363–69. 15157121 10.2165/00007256-200434060-00003

